# Insulin-degrading enzyme ablation in mouse pancreatic alpha cells triggers cell proliferation, hyperplasia and glucagon secretion dysregulation

**DOI:** 10.1007/s00125-022-05729-y

**Published:** 2022-06-02

**Authors:** Beatriz Merino, Elena Casanueva-Álvarez, Iván Quesada, Carlos M. González-Casimiro, Cristina M. Fernández-Díaz, Tamara Postigo-Casado, Malcolm A. Leissring, Klaus H. Kaestner, Germán Perdomo, Irene Cózar-Castellano

**Affiliations:** 1 Unidad de Excelencia Instituto de Biología y Genética Molecular (University of Valladolid-CSIC), Valladolid, Spain; 2 Instituto de Investigación, Desarrollo e Innovación en Biotecnología Sanitaria de Elche (IDiBE), Universidad Miguel Hernández de Elche, Elche, Spain; 3 Centro de Investigación Biomédica en Red de Diabetes y Enfermedades Metabólicas Asociadas (CIBERDEM), Madrid, Spain; 4 IMDEA-Food Institute, CEI UAM+CSIC, Madrid, Spain; 5 Institute for Memory Impairments and Neurological Disorders, University of California, Irvine (UCI MIND), Irvine, CA USA; 6 Department of Genetics and Institute for Diabetes, Obesity and Metabolism, University of Pennsylvania, Philadelphia, PA USA

**Keywords:** Alpha cells, Cytoskeleton, Hyperglucagonaemia, Insulin-degrading enzyme, Primary cilia, Proliferation, Type 2 diabetes

## Abstract

**Aims/hypothesis:**

Type 2 diabetes is characterised by hyperglucagonaemia and perturbed function of pancreatic glucagon-secreting alpha cells but the molecular mechanisms contributing to these phenotypes are poorly understood. Insulin-degrading enzyme (IDE) is present within all islet cells, mostly in alpha cells, in both mice and humans. Furthermore, IDE can degrade glucagon as well as insulin, suggesting that IDE may play an important role in alpha cell function in vivo.

**Methods:**

We have generated and characterised a novel mouse model with alpha cell-specific deletion of *Ide*, the A-IDE-KO mouse line. Glucose metabolism and glucagon secretion in vivo was characterised; isolated islets were tested for glucagon and insulin secretion; alpha cell mass, alpha cell proliferation and α-synuclein levels were determined in pancreas sections by immunostaining.

**Results:**

Targeted deletion of *Ide* exclusively in alpha cells triggers hyperglucagonaemia and alpha cell hyperplasia, resulting in elevated constitutive glucagon secretion. The hyperglucagonaemia is attributable in part to dysregulation of glucagon secretion, specifically an impaired ability of IDE-deficient alpha cells to suppress glucagon release in the presence of high glucose or insulin. IDE deficiency also leads to α-synuclein aggregation in alpha cells, which may contribute to impaired glucagon secretion via cytoskeletal dysfunction. We showed further that IDE deficiency triggers impairments in cilia formation, inducing alpha cell hyperplasia and possibly also contributing to dysregulated glucagon secretion and hyperglucagonaemia.

**Conclusions/interpretation:**

We propose that loss of IDE function in alpha cells contributes to hyperglucagonaemia in type 2 diabetes.

**Graphical abstract:**

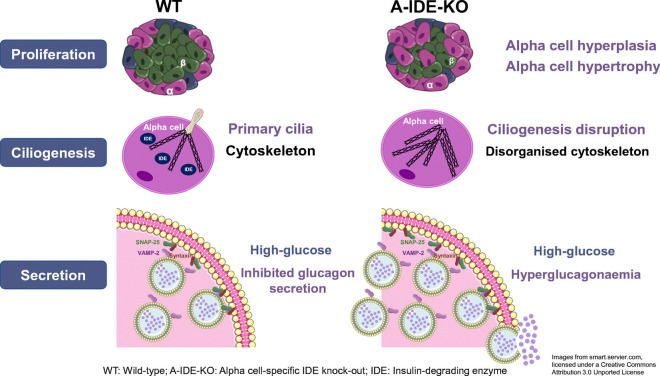

**Supplementary Information:**

The online version contains peer-reviewed but unedited supplementary material available at 10.1007/s00125-022-05729-y.

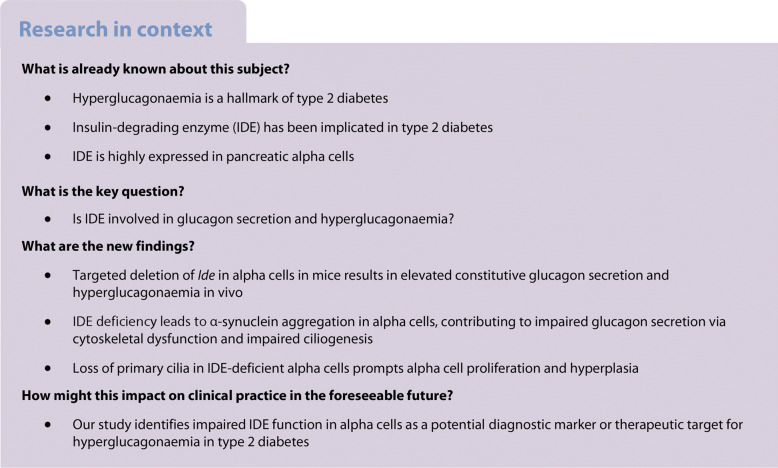



## Introduction

Type 2 diabetes is characterised by hyperglucagonaemia and a decrease in the pancreatic beta/alpha cell ratio [1, 2]. Alpha cell hyperplasia and/or hypertrophy has been postulated to be a principal cause of decreased beta/alpha cell ratio, yet the operative cellular and molecular mechanisms remain poorly understood. Although some evidence points to beta-to-alpha cell transdifferentiation and/or increased alpha cell proliferation as possible causes of hyperglucagonaemia [3, 4], these results remain controversial and need further study.

Genome-wide association studies have identified a locus on chromosome 10 linked to risk for type 2 diabetes. This chromosomal region includes the *Ide* gene that codes for the insulin-degrading enzyme (IDE) [5]*.* Supporting a causal role for IDE, several *Ide* polymorphisms have been identified that are linked to type 2 diabetes risk [6]. Moreover, the Goto–Kakizaki (GK) rat was shown to contain two coding mutations within the *Ide* gene [7]. As a protease that avidly degrades insulin in vitro, IDE had been expected to mediate insulin clearance in vivo; however, emerging evidence has identified several non-proteolytic functions that might be perturbed in type 2 diabetes, implying a complex functional role for IDE [8]. For instance, although pancellular genetic deletion of *Ide* in mice consistently produces a pronounced diabetic phenotype [9, 10], this phenotype has variously been associated with hyperinsulinaemia [9] or hypoinsulinaemia [10]. Liver-specific deletion of *Ide* results in decreased insulin sensitivity but does not impair insulin clearance [11, 12]. Of special interest, in studies of *Ide*-null mice, a key role for IDE in the regulation of insulin secretion from beta cells was identified [10]. Genetic deletion of *Ide* exclusively in beta cells produced impaired glucose-stimulated insulin secretion, along with elevated basal constitutive insulin secretion, decreased cell surface-associated levels of GLUT2, and a phenotype of beta cell functional immaturity [13]. These findings, among many others [8], suggest that IDE is a pleiotropic protein whose involvement in the pathogenesis of type 2 diabetes is likely to be multifaceted.

IDE is present within all islet cells but it is expressed at higher levels in alpha cells from both mice and humans [2]. This, combined with the fact that IDE can degrade glucagon as well as insulin [14], suggests that IDE may play an important role in alpha cell function and/or glucagon homeostasis in vivo. To investigate this topic, we generated a novel mouse line with alpha cell-specific deletion of *Ide*, the A-IDE-KO mouse model, and we performed in vivo and in vitro studies to understand the implication of IDE in alpha cell function and proliferation.

## Methods

### A-IDE-KO (*Gcg-Cre*^ERT2^; *Ide*^f/f^) mouse model

Animal experiments were approved by the University of Valladolid Research Animal Ethical Committee and JCyL regional authorities (protocol no. 5003931) in accordance with European Guidelines for Care and Use of Mammals in Research. Mouse model generation is explained in the electronic supplementary material (ESM) Methods and primers used for genotyping are included in ESM Table 1. All mice were on the C57BL/6J background.

### Plasma biochemistry

Fasting (16 h) blood glucose levels were measured from tail blood using the Contour NEXT Glucometer (Bayer, Germany). Plasma samples were obtained from tail blood after fasting or following glucose administration using blood collection tubes treated with EDTA (Sarstedt, Germany). Plasma glucagon and insulin levels were assessed using mouse ELISA assays (Mercodia, Sweden and Crystal Chem, USA, respectively). Plasma l-amino acids were measured using l-Amino Acid Quantitation Kit (Sigma, USA). See ESM Table 2 for further details.

### IPGTT

To evaluate alterations in glucose homeostasis, we performed an IPGTT. Mice were fasted for 16 h and then injected intraperitoneally with 2 g glucose/kg body weight. Blood glucose levels were quantified immediately before and 15, 30, 60 and 120 min after glucose challenge.

### In vivo glucagon secretion test

Using the same technique as for the IPGTT, blood samples from fasted mice were obtained 0, 5, 15 and 30 min after glucose challenge using blood collection tubes. Plasma was obtained by centrifuging the blood at 3300 *g* for 10 min at 4°C. Glucagon levels were determined by ELISA. See ESM Table 2 for further details.

### Islet isolation and in vitro glucagon and insulin secretion

A-IDE-KO islets were isolated and then recovered in ‘isolation buffer’ for 2 h in an incubator. Afterwards, groups of ten islets of similar size were transferred to 500 μl of ‘secretion buffer’ supplemented with 3 mmol/l glucose for 1 h at 37°C. Next, islet groups were incubated first in 1 mmol/l glucose secretion buffer for 1 h, and afterwards in 16 mmol/l glucose secretion buffer for 1 h. The extracellular medium was collected after each incubation, and glucagon and insulin concentration were measured by ELISA.

To determine pancreas glucagon and insulin content, whole pancreas was incubated overnight in acid-ethanol buffer and hormones were measured in the supernatant fraction by ELISA. See ESM Methods and ESM Table 2 for further details.

### Flow cytometry analysis in isolated islet cells

Flow cytometry analysis (FACS) was used to confirm *Ide* specific ablation in pancreatic alpha cells from the A-IDE-KO model. See ESM Methods for details.

### Ca^2+^ signalling experiments

To study alpha cell function in the A-IDE-KO model, Ca^2+^ signalling patterns were studied in isolated islets. See ESM Methods for details.

### RNA isolation and qRT-PCR

RNA extraction and reverse transcription quantitative real-time PCR (qRT-PCR) were performed as previously described [13]. See ESM Table 1 for primers and TaqMan assays.

### Pancreas histomorphometry

Pancreas histomorphometry was performed as previously described [13]. For measurement of alpha cell mass, glucagon staining was performed by one researcher who took pictures at the microscope and named them ‘blind’; these pictures were then blindly counted by a different researcher. See ESM Methods and ESM Table 2 for further details.

### Pancreas immunostaining

A-IDE-KO and control mouse pancreas sections were stained with the following antibodies diluted in blocking solution (1% BSA, 0.2% normal goat serum in PBS): anti-glucagon (Abcam, UK), anti-IDE (Millipore, USA), anti-Ki67 (Invitrogen, USA), anti-vesicle-associated membrane protein 2 (VAMP-2) (Cell Signaling, USA) and anti-α-synuclein (Santa Cruz Biotechnology, USA). Secondary antibodies were incubated in blocking solution as well. See ESM Table 2 for further details. Immunofluorescence intensity quantification of IDE, VAMP-2 and α-synuclein were performed as previously described [13].

See ESM Methods for further details.

### Alpha-TC-1.9 culture, siRNA experiments and proliferation studies

To quantify proliferation rates, cells were seeded on coverslips and incubated with 10 μmol/l bromodeoxyuridine (BrdU) for 6 h. Staining was performed using monoclonal anti-BrdU rat antibody (Abcam). BrdU staining was performed by one researcher who took pictures at the microscope and named them ‘blind’; these pictures were then blindly counted by a different researcher. BrdU-positive cells were quantified using Image J 1.52p (NIH, USA). To detect the presence of primary cilia, staining was performed using anti-α-acetylated tubulin (Sigma) antibody. Ciliated cells were quantified using Image J 1.52p. See ESM Methods and ESM Table 2 for further details.

### Liver glucagon signalling

For analysis of hepatic glucagon signalling, A-IDE-KO and control mice were fasted for 6 h then administered glucagon (100 μg/kg, i.p.) to activate the pathway. Following euthanasia, livers were dissected, immediately frozen in liquid nitrogen and subsequently stored at −80°C.

### Western blotting

Proteins were extracted from A-IDE-KO and control mouse livers, and from siRNA-CTL and siRNA-*Ide* alpha-TC1.9 cells. Western blots were performed as previously described [13]. See ESM Methods and ESM Table 2 for further details.

### IQR method for outlier recognition

We have applied the interquartile rule to find outliers (IQR method) for identifying outlier values in the qRT-PCRs. See ESM Methods for details.

### Statistics

Randomisation was not performed in the research carried out in this study. All data were included in the manuscript, and no results have been omitted. Statistical analysis was performed using Prism v.4.0 (GraphPad Software, USA). Normality of data was checked with the Kolmogorov–Smirnov test. Data are presented as means ± SEM. Comparisons between two groups were done using two-tailed Student’s *t* test. Comparisons between more than two groups were done using ANOVA. Differences were considered significant at *p*<0.05.

## Results

### A-IDE-KO mice develop hyperglucagonaemia and hyperinsulinaemia causing hepatic glucagon resistance

**Fig. 1:**
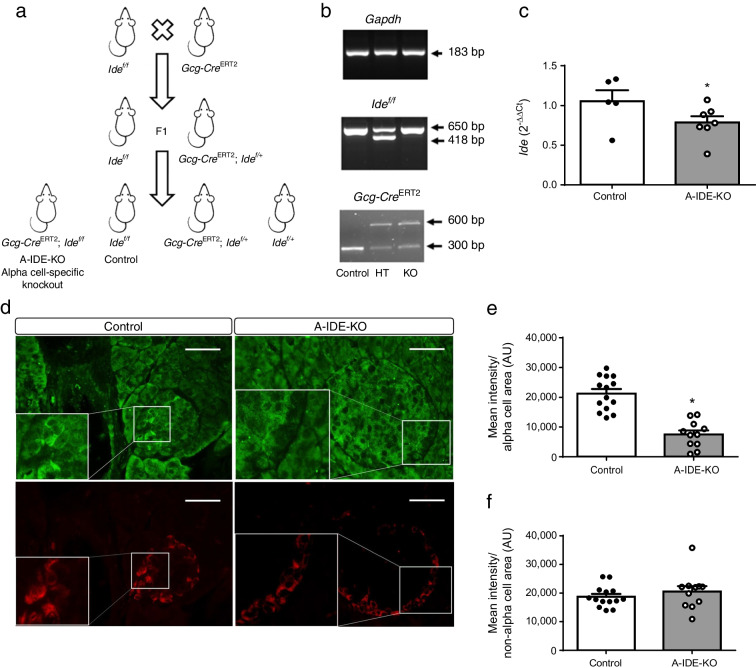
Selective deletion of *Ide* in alpha cells in the A-IDE-KO mouse model. (**a**) Breeding strategy to obtain the A-IDE-KO mouse model. (**b**) Genotyping example showing PCRs for floxed *Ide* and *Gcg-Cre*. Three genotypes are shown: control (*Ide*^f/f^); heterozygous (*Gcg-Cre*^ERT2^; *Ide*^f/+^); and knockout A-IDE-KO (*Gcg-Cre*^ERT2^; *Ide*^f/f^). (**c**) *Ide* expression in A-IDE-KO isolated islets measured by RT-PCR. (**d**–**f**) IDE staining (**d**, representative image) and quantification in alpha (**e**) and non-alpha cell area (**f**) of glucagon (red) and IDE (green) in control (*Ide*^f/f^) and A-IDE-KO (*Gcg-Cre*^ERT2^; *Ide*^f/f^) mouse pancreases. Scale bar, 40 μm. Data are presented as means ± SEM. *p<0.05. AU, arbitrary units; HT, heterozygous; KO, knockout

We have shown previously that IDE is expressed in all islet cells but primarily in alpha cells [2]. To elucidate the role of IDE in alpha cell physiology, an alpha cell-specific *Ide*-knockout mouse model (*Gcg-Cre*^ERT2^; *Ide*^f/f^ [A-IDE-KO]) was generated by breeding mice homozygous for a floxed *Ide* allele (*Ide*^f/f^) [12, 13] with transgenic mice expressing Cre recombinase under the glucagon promoter (*Gcg-Cre*^ERT2^), thus targeting expression to alpha cells [15]. This model is inducible by tamoxifen administration. Strategy for breeding and genotyping control (*Ide*^f/f^), IDE-heterozygous (*Gcg-Cre*^ERT2^; *Ide*^f/+^, not characterised in this manuscript) and IDE-knockout (*Gcg-Cre*^ERT2^; *Ide*^f/f^) mice are shown in Fig. 1a,b.

To verify *Ide* specific ablation in pancreatic alpha cells, we checked *Ide* mRNA expression in islets isolated from A-IDE-KO and control mice and showed a reduction of ~25% in A-IDE-KO mouse islets (corresponding to the percentage of alpha cell population in the islets) (Fig. 1c). Furthermore, we performed IDE and glucagon double immunostaining in A-IDE-KO and control mouse pancreases obtained 8 weeks after tamoxifen treatment. We confirmed IDE-KO specificity in alpha cells, quantifying IDE staining in alpha and non-alpha cell area (Fig. 1d–f). IDE staining was 80% reduced in alpha cells (Fig. 1e) and not changed in the non-alpha cell population (Fig. 1f) of A-IDE-KO mice vs control mice. To confirm this point, we performed FACS analysis to distinguish populations of glucagon^+^IDE^+^ and glucagon^+^IDE^−^ cells, identifying that most alpha cells in A-IDE-KO mouse islets were negative for IDE (ESM Fig. 1). We also performed RT-PCR to study *Ide* expression in tissues involved in glucose metabolism (ESM Fig. 2), showing no changes between A-IDE-KO and control mice. These results nicely support IDE-KO specificity for alpha cells.

**Fig. 2:**
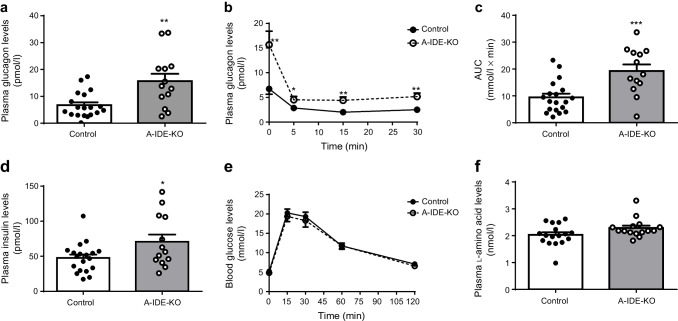
A-IDE-KO mice develop hyperglucagonaemia and hyperinsulinaemia. (**a**) Fasting glucagon levels. (**b**, **c**) Glucagon secretion in vivo after glucose challenge (**b**) and the corresponding AUC (**c**), *n* = 19 control and *n* = 13 A-IDE-KO. (**d**) Fasting plasma insulin levels. (**e**) IPGTT, *n* = 19 control and *n* = 11 A-IDE-KO. (**f**) l-Amino acids in circulation. *n* = 11–19 mice per group. Data are presented as means ± SEM. *p<0.05, **p<0.01 and ***p<0.001 vs control

We performed metabolic characterisation of male and female A-IDE-KO mice 1 month after tamoxifen treatment under fasting conditions (16 h). Relative to control mice, A-IDE-KO mice exhibited significant hyperglucagonaemia both in the fasting state (Fig. 2a) and after glucose overload (Fig. 2b,c). A-IDE-KO mice also exhibited significantly elevated fasting plasma insulin levels (Fig. 2d) but, unexpectedly, did not differ from control mice in IPGTT results (Fig. 2e). It has been recently reported that l-amino acids are increased in the circulation after impaired hepatic glucagon signalling, and this is especially relevant because raised levels of these amino acids have been shown to induce alpha cell proliferation [16]. However, the plasma l-amino acid levels in A-IDE-KO mice did not differ from the levels in control mice (Fig. 2f).

**Fig. 3:**
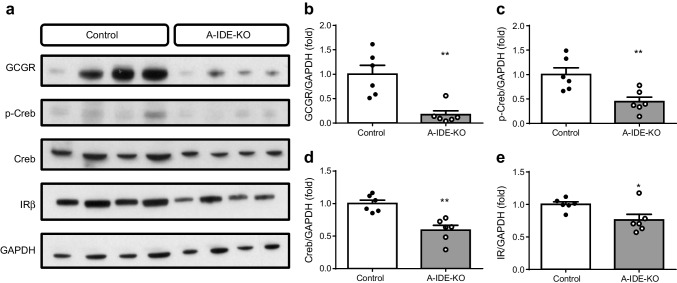
Hepatic glucagon resistance in A-IDE-KO mice. (**a**) Representative western blot of glucagon signalling in glucagon-stimulated liver extracts from control and A-IDE-KO mice. (**b**) Glucagon receptor quantification. (**c**) p-Creb quantification. (**d**) Creb quantification. (**e**) Insulin receptor quantification. *n* = 6 mice per group. Data are presented as means ± SEM. *p<0.05 and **p<0.01 vs control. GCGR, glucagon receptor; IR insulin receptor

To better characterise glucagon signalling, mice were injected with 100 μg/kg glucagon, then killed 10 min later, whereupon the liver was removed for analysis. We found a significant decrease in glucagon receptor (Fig. 3a,b), p-Creb (Fig. 3a,c) and Creb levels (Fig. 3a,d), pointing to hepatic glucagon resistance (Fig. 3a–c). Interestingly, insulin receptor levels were decreased in A-IDE-KO mouse livers, as well (Fig. 3e). These data help to explain the absence of glucose intolerance in the presence of hyperglucagonaemia.

### A-IDE-KO mice show increased alpha cell mass and constitutive glucagon secretion

**Fig. 4:**
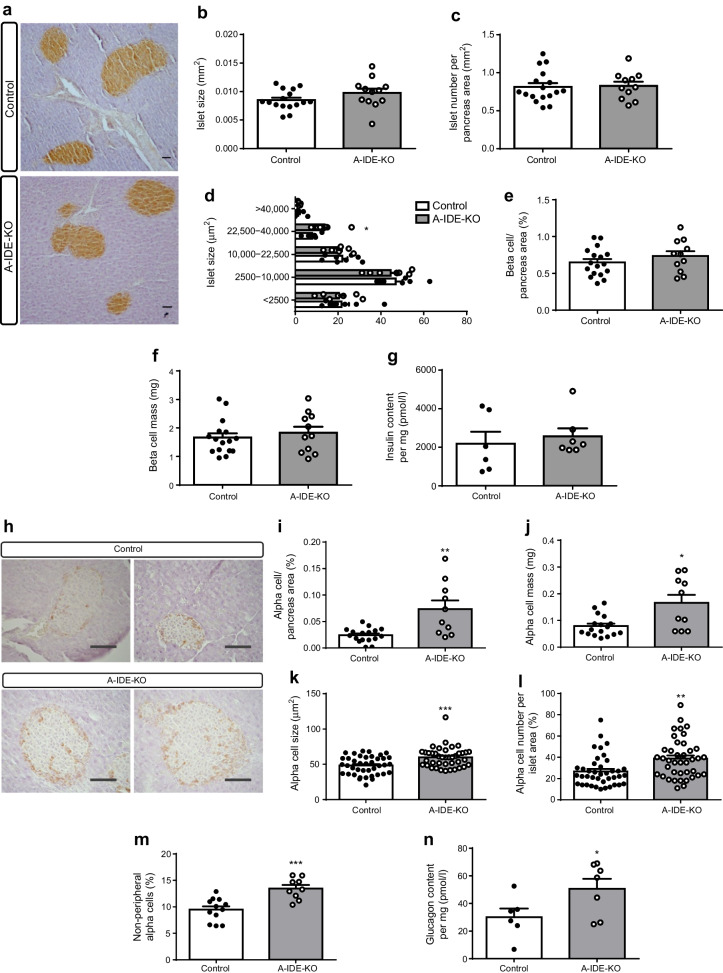
A-IDE-KO mouse pancreases exhibit increased population of large islets, increased alpha cell mass and more non-peripheral alpha cells. (**a**) Representative immunohistochemistry of insulin in pancreatic tissue. Scale bar, 40 μm. (**b**) Islet size. (**c**) Islet number per pancreas area. (**d**) Islet size distribution analysis. (**e**) Beta cell area. (**f**) Beta cell mass. (**g**) Intrapancreatic insulin content obtained by acid-ethanol extraction and measured by ELISA. (**h**) Representative immunohistochemistry of glucagon in pancreas tissue. Scale bar, 40 μm. (**i**) Alpha cell area per pancreas area. (**j**) Alpha cell mass. (**k**) Alpha cell size. (**l**) Non-peripheral alpha cell number. (**m**) Non-peripheral alpha cells. (**n**) Intrapancreatic glucagon content obtained by acid-ethanol extraction and measured by ELISA. *n* = 6–16 mice per group. Data are presented as means ± SEM. *p<0.05, **p<0.01 and ***p<0.001 vs control

To unravel the causes of hyperglucagonaemia and hyperinsulinaemia in this mouse model, we studied pancreas histomorphometry. Insulin staining revealed normal beta cell mass (Fig. 4a–f) and higher numbers of large islets in A-IDE-KO mouse pancreases (Fig. 4d). Normal beta cell mass in the A-IDE-KO (vs control) mice was confirmed by quantification of intrapancreatic insulin (Fig. 4g). Interestingly, alpha cell mass was increased in A-IDE-KO mouse pancreases, and glucagon staining revealed larger and more numerous alpha cells (hypertrophy and hyperplasia) (Fig. 4h–l). The number of non-peripheral alpha cells, a signature of islet dysfunction, was also increased in A-IDE-KO mice (Fig. 4m). Consistent with increased alpha cell mass, intrapancreatic glucagon levels were elevated in A-IDE-KO mouse pancreases (Fig. 4n).

**Fig. 5:**
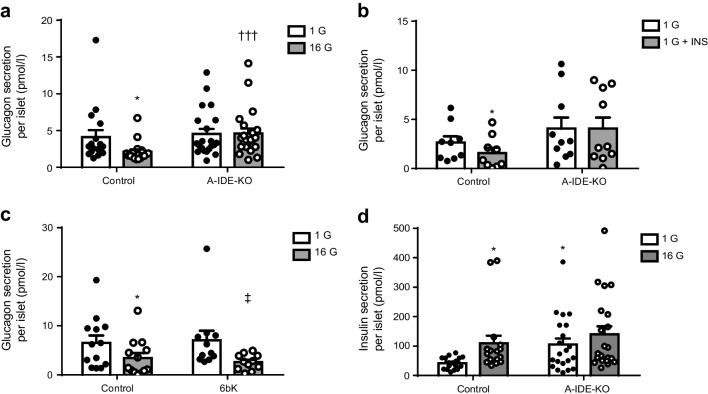
Isolated A-IDE-KO islets show constitutive glucagon and insulin secretion. (**a**) Glucagon secreted by islets, isolated from control and A-IDE-KO mice, in response to low and high glucose (1 mmol/l glucose; 16 mmol/l glucose). (**b**) Glucagon secreted by isolated islets in response to insulin inhibition (in the presence of 1 mmol/l glucose). (**c**) Glucagon secreted by wild-type C57Bl/6J mouse islets treated with 10 μmol/l 6bK (selective IDE inhibitor) for 30 min, in response to low and high glucose. (**d**) Insulin secreted by isolated islets in response to low and high glucose. *n* = 17–21 islet batches. Data are presented as means ± SEM. *p<0.05 vs control 1 mmol/l glucose; ^†††^*p*<0.001 vs control 16 mmol/l glucose; ^‡^*p*<0.05 vs A-IDE-KO 16 mmol/l glucose. G, mmol/l glucose; INS, insulin

To understand how IDE affects alpha cell function, we studied glucagon secretion ex vivo in islets isolated from A-IDE-KO and control mice. As expected, control islets showed decreased glucagon secretion when exposed to high glucose levels; in marked contrast, A-IDE-KO islets failed to inhibit glucagon secretion in the presence of high glucose (Fig. 5a). Similarly, whereas insulin inhibited glucagon secretion, no such inhibition occurred in A-IDE-KO islets (Fig. 5b). These data suggest that IDE is essential for the regulation of stimulated glucagon secretion in alpha cells. Interestingly, IDE pharmacological inhibition by the specific inhibitor 6bK produced no changes in glucose-stimulated glucagon secretion (Fig. 5c). This result points to a non-proteolytical function of IDE in the A-IDE-KO model.

In parallel, insulin secretion in A-IDE-KO mouse islets was also perturbed, with the islets exhibiting constitutive insulin secretion compared with control mouse islets, secreting insulin independently of glucose concentration (Fig. 5d).

Although glucagon secretion is a complex process regulated by several intracellular signals [17], alpha cell exocytosis is a Ca^2+^-sensitive process. Hence, we examined whether the lack of suppression of glucagon secretion at 16 mmol/l glucose in A-IDE-KO mouse islets was associated with abnormal Ca^2+^ levels. As shown in ESM Fig. 3, glucose induced similar Ca^2+^ signalling patterns in alpha cells from both control and A-IDE-KO mouse islets. These results suggest that effects led by decreased IDE expression on glucagon secretion occur downstream of Ca^2+^ signalling.

### Alpha cell identity and exocytosis machinery are modified in absence of IDE

**Fig. 6:**
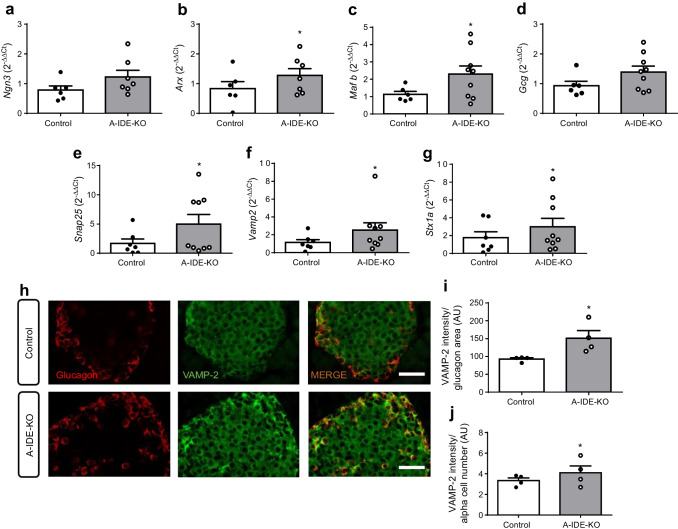
A-IDE-KO mouse islets show increased expression of genes regulating alpha cell fate and SNARE complex. (**a**–**g**) *Ngn3* (**a**), *Arx* (**b**), *Mafb* (**c**), *Gcg* (**d**), *Snap25* (**e**), *Vamp2* (**f**) and *Stx1a* (**g**) mRNA expression measured by quantitative PCR (*n* = 7 [control wild-type mice]; *n* = 9 [A-IDE-KO mice]). (**h**) Representative images of pancreas double immunostaining with glucagon (red), VAMP-2 (green) and overlap of both (orange). Scale bar, 40 μm. (**i**, **j**) VAMP-2 intensity normalised to glucagon area (**i**) and non-glucagon area (**j**). *n* = 4 pancreases per group. Data are presented as means ± SEM. *p<0.05 vs control. AU, arbitrary units

To further elucidate how IDE participates in the regulation of alpha cell identity and differentiation, we analysed the expression of several genes involved in the maintenance of alpha cell physiology in adult pancreas (*Ngn3* [also known as *Neurog3*], *Arx*, *Mafb* and *Gcg*). Interestingly, A-IDE-KO islets showed significant increases in *Arx* and *Mafb*, and non-significant increases in *Ngn3* and *Gcg* (Fig. 6a–d). Because dysregulation of glucagon secretion was observed (Fig. 5), we also quantified the expression of several SNARE family genes, which revealed that *Snap25*, syntaxin 1A and *Vamp2* were significantly elevated (Fig. 6e–g). Increased VAMP-2 protein levels in A-IDE-KO alpha cells was confirmed by VAMP-2–glucagon double labelling and quantification (Fig. 6h–j). These findings provide additional evidence that alpha cell function is altered by deletion of *Ide*.

### *Ide* ablation induces alpha cell proliferation due to cytoskeleton dysregulation and impaired ciliogenesis

**Fig. 7:**
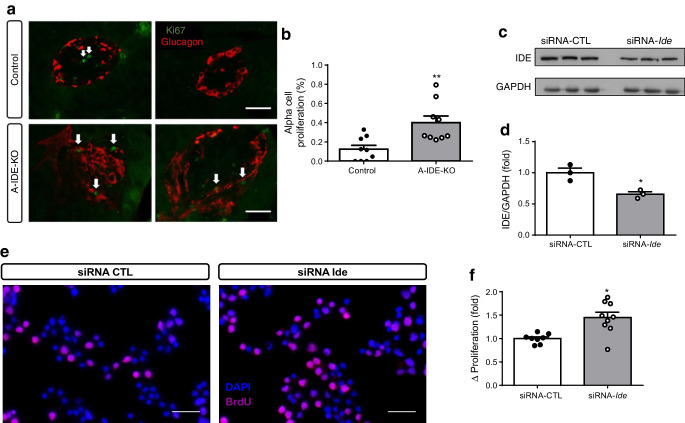
Deletion of IDE triggers alpha cell proliferation. (**a**) Representative images of Ki67 (green) and glucagon (red) staining in A-IDE-KO and control mouse pancreases. Scale bar, 40 μm. Arrows point to proliferative/Ki67-positive cells. (**b**) Quantification of alpha cell proliferation by Ki67/glucagon cells per total number of glucagon cells (*n* = 9). (**c**, **d**) IDE-knockdown in alpha-TC1.9 cells using siRNA-*Ide* or siRNA-CTL (scrambled control), showing a ~40% decrease in IDE expression (*n* = 3). (**e**) Representative images of BrdU staining in IDE-deficient and control alpha-TC1.9 cells. Scale bar, 100 μm. (**f**) Quantification of proliferation by detection of BrdU-positive cells (*n* = 9). Data are presented as means ± SEM. *p<0.05 and **p<0.01 vs control mouse or vs siRNA-CTL treatment

To investigate whether increased cell proliferation was the cause of alpha cell hyperplasia, we immunostained A-IDE-KO and control mouse pancreases for Ki67 and glucagon. Ki67-positive alpha cells were increased approximately fourfold in A-IDE-KO mouse pancreas relative to control mouse pancreas (Fig. 7a,b). To confirm that increased proliferation was directly attributable to loss of *Ide* expression, we used siRNA to knockdown IDE levels by ~40% in the alpha cell line alpha-TC1.9 (Fig. 7c,d). The cells with reduced IDE expression proliferated 50% more than controls (siRNA-CTL-treated), as quantified by BrdU staining (Fig. 7e,f).

**Fig. 8:**
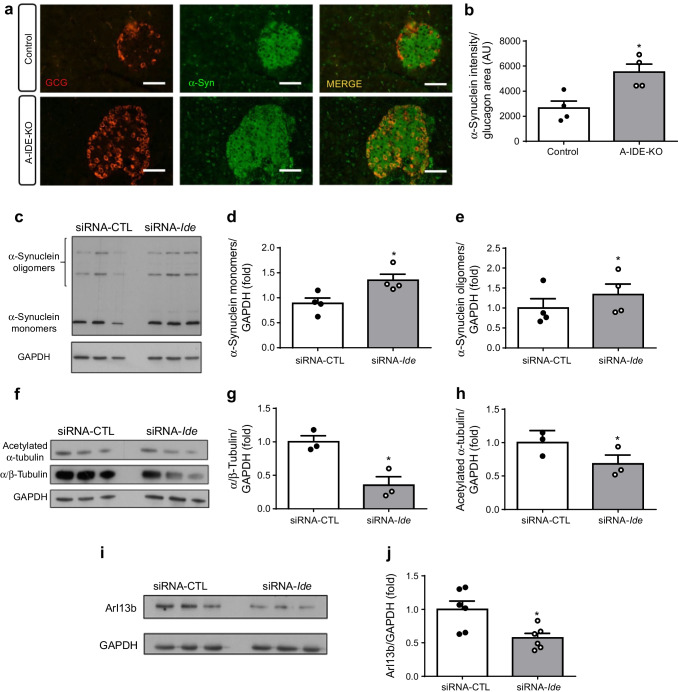

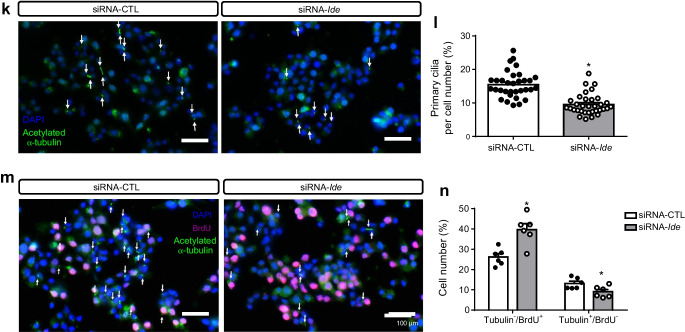
Loss of IDE expression triggers increased α-synuclein oligomer formation, impaired ciliogenesis and cytoskeletal defects. (**a**) Representative images of α-synuclein staining in A-IDE-KO and control mouse pancreases. Scale bar, 40 μm. (**b**) Quantification of α-synuclein staining in glucagon-positive cells (*n* = 4). (**c**) Representative western blot of α-synuclein monomers and oligomers in siRNA-*Ide*- and siRNA-CTL (scrambled control)-treated alpha-TC1.9 cells. (**d**, **e**) Quantification of α-synuclein by western blotting, showing monomers (**d**) and oligomers (**e**) (*n* = 4). (**f**) Representative western blot of α/β-tubulin and acetylated α-tubulin in siRNA-*Ide*- and siRNA-CTL-treated alpha-TC1.9 cells. (**g**) Quantification of α/β-tubulin levels by western blot (*n* = 3). (**h**) Quantification of acetylated α-tubulin by western blotting (*n* = 3). (**i**) Representative western blot of Arl13b, a cilia-specific GTPase, in siRNA-*Ide*- and siRNA-CTL-treated alpha-TC1.9 cells. (**j**) Quantification of Arl13b by western blotting (*n* = 6). (**k**) Representative images of ciliated alpha cells stained for acetylated α-tubulin in siRNA-*Ide*- and siRNA-CTL-treated alpha-TC1.9 cells. Scale bar, 100 μm. Arrows point to ciliated cells. (**l**) Quantification of ciliated cells in siRNA-*Ide*- and siRNA-CTL-treated alpha-TC1.9 cells (*n* = 9). (**m**) Representative images of proliferating vs non-proliferating ciliated alpha cells in siRNA-*Ide*- and siRNA-CTL-treated alpha-TC1.9 cells. Scale bar, 100 μm. Arrows point to ciliated cells. (**n**) Quantification of BrdU per cilium in siRNA-*Ide*- and siRNA-CTL-treated alpha-TC1.9 cells (*n* = 6). Data are presented as means ± SEM. *p<0.05 vs control mouse or siRNA-CTL treatment. AU, arbitrary units; GCG, glucagon; α-Syn, α-synuclein

In searching for a mechanism to explain the increased alpha cell proliferation and the perturbation in stimulated release of glucagon and insulin, we elected to focus on α-synuclein, a small, aggregation-prone protein that has been shown to regulate the cytoskeleton in several secretory cell types, including neurons and endocrine cells [18]. IDE regulates intracellular levels of α-synuclein as well as its aggregation by a non-proteolytic interaction wherein α-synuclein monomers bind essentially irreversibly to IDE. In total-IDE-knockout beta cells, which show impairment in glucose-stimulated insulin secretion, levels of α-synuclein were found to be elevated, in association with reduction in the releasable pool of insulin granules, disruption in autophagic flux and diminished microtubule content [10]. Supporting a similar functional role for IDE in alpha cells, α-synuclein levels were found to be elevated by ~100% and ~40% in A-IDE-KO mouse pancreases and IDE-knockdown cells, respectively, relative to respective controls (Fig. 8a–e). Also consistent with the known role of α-synuclein in regulating the cytoskeleton [10], levels of α/β-tubulin were diminished by ~60% in IDE-knockdown alpha-TC1.9 cells (Fig. 8f,g).

Disruptions to the cytoskeleton can also impede the development and functioning of cilia, which play a role in secretory processes [19, 20]. Consistent with a role for IDE in regulating cytoskeletal function, IDE-knockdown cells showed a ~30% decrease in acetylated α-tubulin, a marker of tubulin polymerisation required for cilia formation and stability [21] (Fig. 8f,h) and a ~50% decrease in Arl13b, a small GTPase localised within cilia [22] (Fig. 8i,j). Furthermore, IDE-knockdown alpha-TC1.9 cells (Fig. 8k,l) exhibited reduced cilia number relative to control cells, demonstrated by reduced punctate acetylated α-tubulin immunoreactivity. It has been shown in several cell types that proliferation capacity diminishes once the primary cilia are completely formed and, conversely, that cell proliferation increases in their absence [23]. If cilia provide information that serves to retain cells in their functional, differentiated G_0_ state, then, defects in this pathway are predicted to cause proliferative disorders [23]. To address whether *Ide* loss could disrupt cell-cycle entry, we double-labelled cells with acetylated α-tubulin and anti-BrdU antibodies to determine whether or not proliferative alpha cells are ciliated. Most proliferative cells were found to be non-ciliated and, remarkably, non-ciliated proliferative cells were augmented in IDE-deficient alpha-TC1.9 cells compared with control cells (Fig. 8m,n). These results suggest that *Ide* loss enhances alpha cell proliferation by diminishing ciliogenesis.

## Discussion

IDE has been implicated in the pathogenesis of type 2 diabetes but its role in different tissues involved in glucose homeostasis has only recently begun to be elucidated [11–13, 24]. To clarify the role of IDE in alpha cell function, we developed a novel alpha cell-specific *Ide*-knockout mouse model (A-IDE-KO). Deletion of *Ide* in alpha cells resulted in a metabolic phenotype consisting of hyperglucagonaemia and hyperinsulinaemia but with normal glucose tolerance. The hyperinsulinaemia is likely due to an exacerbated paracrine effect wherein excess glucagon release by alpha cells stimulates the glucagon receptors on beta cells, leading to activation of the cAMP– protein kinase A (PKA)–exchange protein directly activated by cAMP (EPAC) pathway and thereby stimulating insulin secretion [25–28]. Alternatively, or in addition, this phenotype could be attributed to dysregulated paracrine control as a consequence of impaired alpha cell ciliogenesis, as previously reported for beta cell paracrine control in the beta cell-specific IFT88-KO model [22]. In this model it was shown that the response of beta cells to somatostatin is dependent on the cilia, consistent with the fact that the somatostatin receptor 3 (SSTR3) localises to beta cell cilia. It was also observed that global changes occurred in modulation of pathways governing paracrine signalling, hormone secretion, islet cell connectivity and calcium activation [22].

Supporting this, A-IDE-KO mice have normal beta cell mass and display no beta cell hypertrophy or hyperplasia. The hyperglucagonaemia in A-IDE-KO mice appears to be a primary phenotype produced by a constellation of underlying causes, including augmented alpha cell mass (attributable to alpha cell hyperplasia and hypertrophy) and dysregulation of glucagon secretion. In particular, high glucose failed to inhibit glucagon secretion in A-IDE-KO mouse islets. Similarly, A-IDE-KO mouse islets were unresponsive to the normal inhibitory effect of insulin. Together, these two effects result in a phenotype of constitutively elevated glucagon secretion, closely paralleling the phenotype of constitutive insulin secretion produced by deletion of *Ide* from beta cells [2]. The fact that the common consequence of *Ide* deletion in both alpha and beta cells is dysregulation of hormone secretion strongly supports the idea that IDE plays an important functional role in secretory processes [10, 13]. It is noteworthy that the proteolytic function of IDE does not seem to play a significant role in the regulation of these peptide hormones in vivo as was once assumed.

Surprisingly, A-IDE-KO mice display normal fasting glucose levels and physiological glucose tolerance, apparently attributable to liver glucagon resistance in the form of reduced glucagon signalling. This may represent a compensatory response to chronic exposure of hepatocytes to hyperglucagonaemia that prompts a reduction in glucagon receptor levels and impaired p-Creb/Creb signalling. Glucagon resistance would be circumventing hepatic glucose production and hyperglycaemia in this preclinical model [29].

There are differences in the effects of IDE deletion on glucagon secretion in vivo vs ex vivo. Although glucagon secretion was increased in both paradigms, isolated islets showed constitutive glucagon secretion that was not inhibited by high glucose or insulin. By contrast, in vivo glucagon levels were significantly attenuated at 5 min after glucose challenge. Two factors may help to account for this discrepancy. First, plasma glucagon levels reflect the balance between glucagon secretion and its clearance in vivo [30] but clearance mechanisms are not present ex vivo. Second, an important mechanism for in vivo glucagon secretion is hypoglycaemia-induced activity of the pancreatic innervation [31], which is not operative in isolated islets.

At the level of gene expression, islets isolated from A-IDE-KO mice expressed increased levels of *Arx* and *Mafb* relative to control islets. *Arx* is required for alpha cell development, promoting their specialisation and differentiation, and overexpression of this transcription factor has been strongly implicated in alpha cell hyperplasia [32]. *Mafb* is expressed in both alpha and beta cells during endocrine pancreas development [33, 34] but becomes specific to the alpha cell lineage 2 weeks after birth [34]. This transcriptional factor has been described as a key regulator of glucagon gene expression [35]. In alignment with the increase in these two transcription factors, *Gcg* transcripts were non-significantly elevated in A-IDE-KO mouse islets. Deletion of *Ide* also resulted in increased expression of genes coding for several members of the SNARE protein complex, including *Snap25*, *Stx1a* and *Vamp2*. Because the SNARE complex plays a key role in facilitating the fusion of glucagon granules to the plasma membrane, regulating cellular exocytosis, it is reasonable that these genes would be upregulated to meet the demand of continuous glucagon secretion [36].

Histomorphometric studies also revealed that alpha cell number and size were increased in A-IDE-KO mouse pancreases, resulting in greater alpha cell mass. Evidence supporting this phenotype being attributable to augmented cell proliferation was provided by independent studies in cultured alpha cell lines with or without siRNA-mediated knockdown of IDE. It is surprising that ~40% reduction in IDE resulted in a ~50% activation of cell proliferation. Based on published studies, one could argue that this effect might be mediated by an interaction between IDE and the retinoblastoma protein (pRb), a tumour suppressor that inhibits cell-cycle progression at the G_1_/S transition when interacting with E2F transcription factors [37]. IDE co-purifies with pRb on proteasomal preparations of breast cancer and hepatoma cells [38]. Similarly, IDE has been shown to co-immunoprecipitate with the tumour suppressor phosphatase and tensin homologue (PTEN), accelerating its degradation by sirtuin-4 (SIRT4) in response to nutritional starvation stresses [39]. Although the functional significance of these protein–protein interactions remains to be fully elucidated, these findings are consistent with a functional role for IDE in regulating cell proliferation and, possibly, oncogenesis. Interestingly, we observed that proliferating alpha cells exhibit a significant diminution in the abundance of cilia, an important hallmark of alpha cell differentiation with important functions in beta cells and in paracrine islet signals [22]. These findings raise several questions. For example, how precisely do cilia contribute to alpha cell proliferation? Are the effects of IDE deficiency on ciliogenesis specific or perhaps symptomatic of a more general effect on cytoskeletal homeostasis? Could the reduction in cilia abundance contribute to the dysregulation of glucagon and insulin secretion? What precisely is the role of IDE in this connection?

Deletion of *Ide* in alpha cells (this study) and beta cells [10, 13] produces dysregulation of glucagon and insulin secretion, respectively, and in both cases also results in increases in the accumulation of oligomeric α-synuclein. IDE binds avidly to monomeric α-synuclein, leading to the formation of stable and irreversible complexes, thereby slowing the formation of higher-n aggregates of α-synuclein. Steneberg et al postulated that *Ide* deletion impairs insulin secretion from beta cells by promoting aggregation of α-synuclein, which in turn disrupts microtubule function and impairs secretion processes dependent on the integrity of the cytoskeleton [10]. We show here that pancreatic alpha cells also express α-synuclein and that levels of oligomeric α-synuclein species are increased in both A-IDE-KO mouse pancreases and IDE-knockdown cells. The hypothesis that α-synuclein oligomer formation leads to cytoskeletal disorders is corroborated by the present study, which found that alpha cells lacking IDE harbour increased α-synuclein aggregates together with decreased levels of acetylated α-tubulin, which is required for microtubule stabilisation and the assembly of primary cilia [21]. Cilia are microtubule-based structures that protrude from the cell surface and function as sensors for mechanical and chemical ecological cues that regulate cellular differentiation and division [40]. Beta cell cilia are required for normal insulin secretion [20] and it has been reported that beta cell cilia loss affects paracrine interactions in the islet and causes altered glucagon and somatostatin secretion [22]. Notably, Gerdes et al established a link between primary cilia and diabetes in GK rats, finding impaired glucose-stimulated insulin secretion and fewer ciliated beta cells in these animals relative to controls [20]. Interestingly, GK rats harbour loss-of-function mutations in the *Ide* gene [7], resulting in inhibition of IDE’s ability to degrade amyloid peptides [41]. In addition, the dynamics of the microtubule network play an important role in pancreatic beta cell secretion. Microtubule depolymerisation by glucose or pharmacological agents enhances insulin secretion by increasing the incorporation of granules at exocytotic sites [42].

If cilia provide information that serves to retain cells in their functional, differentiated G_0_ state, then defects in this pathway could cause proliferative disorders such as cancer [23]. Furthermore, the absence of cilia has been associated with increased proliferation in several cell types, including beta cells [43]. Thus, impaired ciliogenesis may underlie the increased proliferation in IDE-deficient alpha cells. In another connection, α-synuclein also interacts with the cytoplasmic terminus of Kir6.2, a major subunit of the of ATP-sensitive potassium channel (K_ATP_), common in both beta and alpha cells, inducing impaired insulin secretion [44]. Whether this interaction between α-synuclein and Kir6.2 occurs in alpha cells and contributes to the observed glucagon secretion dysregulation warrants further study.

There is another important question with respect to elevated α-synuclein levels, since it has been previously shown that maintenance of continuous presynaptic SNARE complex assembly requires a nonclassical chaperone activity mediated by synucleins in neurons. More specifically, α-synuclein directly binds to the SNARE protein VAMP-2 and promotes SNARE complex assembly [45]. This evidence may explain elevated levels of SNARE complex proteins in A-IDE-KO mice. Indeed, it has been recently reported that treatment of isolated islets with α-synuclein monomers increases glucose-stimulated insulin secretion [46], suggesting that the effect of α-synuclein on exocytosis also occurs in islet cells.

Our findings indicate that decreased IDE expression in A-IDE-KO mouse islets mainly affected glucagon secretion at steps downstream of Ca^2+^ signals [47–49]. Indeed, several molecules involved in the secretory process, such as SNARE proteins, α-synuclein and microtubules, were altered in the A-IDE-KO model. These changes have been associated with facilitated secretion in the pancreatic beta cell. Comparably, the overall effect of these alterations on the alpha cell secretory process could account for the lack of suppression of glucagon secretion at 16 mmol/l glucose in A-IDE-KO mouse islets, despite decreased alpha cell Ca^2+^ signalling, since the secretory output may depend on the balance of multiple regulatory factors.

In conclusion, selective deletion of *Ide* in alpha cells triggers hyperglucagonaemia and alpha cell hyperplasia, resulting in elevated constitutive glucagon secretion. We propose that loss of IDE expression in alpha cells may contribute to hyperglucagonaemia in type 2 diabetes.

### Supplementary information

ESM 1 (PDF 762 kb)

## Data Availability

All data generated or analysed during this study are included in this published article (and its supplementary information files).

## Abbreviations

BrdU: Bromodeoxyuridine
GK: Goto–Kakizaki
IDE: Insulin-degrading enzyme
pRb: Retinoblastoma protein
VAMP-2: Vesicle-associated membrane protein 2
