# Correction to: Insulin-degrading enzyme ablation in mouse pancreatic alpha cells triggers cell proliferation, hyperplasia and glucagon secretion dysregulation

**DOI:** 10.1007/s00125-023-06037-9

**Published:** 2023-11-15

**Authors:** Beatriz Merino, Elena Casanueva-Álvarez, Iván Quesada, Carlos M. González-Casimiro, Cristina M. Fernández-Díaz, Tamara Postigo-Casado, Malcolm A. Leissring, Klaus H. Kaestner, Germán Perdomo, Irene Cózar-Castellano

**Affiliations:** 1 Unidad de Excelencia Instituto de Biología y Genética Molecular, University of Valladolid-CSIC), Valladolid, Spain; 2 Instituto de Investigación, Desarrollo e Innovación en Biotecnología Sanitaria de Elche (IDiBE), Universidad Miguel Hernández de Elche, Elche, Spain; 3 Centro de Investigación Biomédica en Red de Diabetes y Enfermedades Metabólicas Asociadas (CIBERDEM), Madrid, Spain; 4 IMDEA-Food Institute, CEI UAM+CSIC, Madrid, Spain; 5 Institute for Memory Impairments and Neurological Disorders, University of California, Irvine (UCI MIND), Irvine, CA USA; 6 Department of Genetics and Institute for Diabetes, Obesity and Metabolism, University of Pennsylvania, Philadelphia, PA USA

**Correction to: Diabetologia**

**https://doi.org/10.1007/s00125-022-05729-y**

Unfortunately, in the BrdU staining of alpha-TC1.9 cells shown in Fig. 7e, the representative image used in the siRNA-*Ide* panel was a duplication of the image used for the control panel. The authors assert that this mistake had no impact on the data analysis, interpretation or conclusions drawn. Figure 7e in the original article has been corrected.

**Fig. 7:**
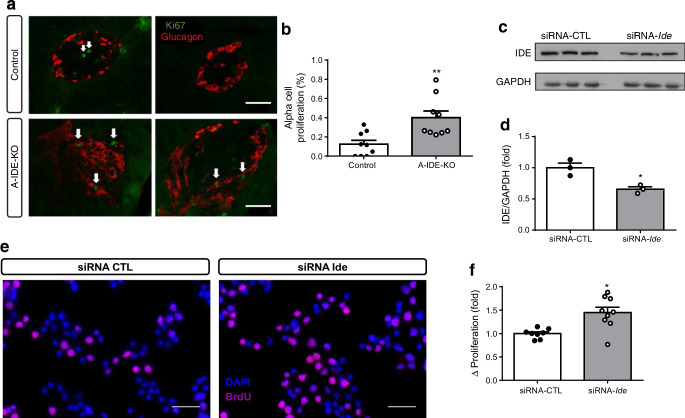
Deletion of IDE triggers alpha cell proliferation. (**a**) Representative images of Ki67 (green) and glucagon (red) staining in A-IDE-KO and control mouse pancreases. Scale bar, 40 μm. Arrows point to proliferative/Ki67-positive cells. (**b**) Quantification of alpha cell proliferation by Ki67/glucagon cells per total number of glucagon cells (*n* = 9). (**c**, **d**) IDE-knockdown in alpha-TC1.9 cells using siRNA-Ide or siRNA-CTL (scrambled control), showing a ~40% decrease in IDE expression (*n* = 3). (**e**) Representative images of BrdU staining in IDE-deficient and control alpha-TC1.9 cells. Scale bar, 100 μm. (**f**) Quantification of proliferation by detection of BrdU-positive cells (*n* = 9). Data are presented as means ± SEM. *p < 0.05 and **p < 0.01 vs control mouse or vs siRNA-CTL treatment

