# Supplementary material for: Insulin-degrading enzyme ablation in mouse pancreatic alpha cells triggers cell proliferation, hyperplasia and glucagon secretion dysregulation
Source: Diabetologia. 2022 Jun 2;65(8):1375–89. doi: 10.1007/s00125-022-05729-y (PMC9283140; doi:10.1007/s00125-022-05729-y)
Supplement: Supplementary file 1 — (PDF 762 kb) [file 125_2022_5729_MOESM1_ESM.pdf]

ESM Methods

A-IDE-KO (*Gcg-Cre*^{ERT2}; *Ide*$^{f/f}$) mouse model

The Cre/LoxP system was used for generating alpha-cell specific IDE knockout mice. Mice homozygous for a “floxed” *Ide* gene (i.e., with loxP sites flanking Exon 3 of the *Ide* gene) (*Ide*$^{f/f}$ mice) [9, 11-13] were crossed to the *Gcg-Cre*^{ERT2} mouse line [16] that expresses Cre recombinase (Cre) fused to a mutant estrogen ligand-binding domain (ER^{T2}), which requires the presence of tamoxifen for activity in pancreatic alpha-cells without disrupting preproglucagon gene expression. Both mouse lines are in the C57BL/6J background. The two lines were bred to obtain mice with two copies of the floxed *Ide* allele together with a single copy of *Gcg-Cre*^{ERT2}. To achieve alpha-cell-specific, Cre-mediated deletion of *Ide*, 5- to 8-week-old *Gcg-Cre* ^{ERT2}; *Ide*$^{f/f}$ mice were injected intraperitoneally with 100 µg/g body weight of tamoxifen (Sigma, USA) in 20 mg/mL corn oil (Sigma, USA) once daily for 3 consecutive days, in order to activate *Ide* silencing. The resulting tamoxifen-injected *Gcg-Cre*^{ERT2}; *Ide*$^{f/f}$ mice are referred to as the A-IDE-KO line; *Ide*$^{f/f}$ and Ide$^{f/+}$ (*Ide*$^{f/f}$) lines were used as controls. Experimental procedures were performed 4-8 weeks post-tamoxifen induction. *Gcg-Cre*^{ERT2}; *Ide*$^{f/f}$ mice without tamoxifen induction did not show abnormal glucagonaemia or glucagon secretion compared to *Ide*$^{f/f}$ mice without tamoxifen (ESM Fig. 4). Mice were fed standard rodent chow diet and water *ad libitum* and housed in ventilated cages under a 12:12h light:dark cycle. For genotyping, PCR was performed with tail DNA isolated using QuickExtract™ DNA Extraction Solution (Epicentre, USA) according with the manufacturer's instructions. *Gapdh, Ide* and *Gcg-Cre*^{ERT2} genes were amplified using the primers described in ESM Table 1.

Islet isolation and *in vitro* glucagon and insulin secretion

A-IDE-KO islets were isolated by pancreatic duct perfusion with Collagenase V (1,000U/mL) (Sigma, USA) in “isolation buffer” (115 mmol/l NaCl; 10 mmol/l $NaHCO_3$; 5 mmol/l KCl; 1.1 mmol/l $MgCl_2$; 25 mmol/l HEPES; 1.2 mmol/l NaH_2PO_4; 2.5 mmol/l $CaCl_2$; 5.5 mmol/l Glucose; 0.1% BSA; pH 7.4). The pancreas was digested in a stationary bath at 37ºC for 14 min. Islets were collected by hand-picking under a stereomicroscope. Freshly isolated islets were left to recover in isolation buffer for 2 h in an incubator. After recovery, groups of 10 islets of similar size were transferred to 500 µl of “secretion buffer” (140 mmol/l NaCl, 4.5 mmol/l KCl, 2.5 mmol/l $CaCl_2$, 1 mmol/l $MgCl_2$, 20 mmol/l HEPES, 0.1% BSA, pH 7.4) supplemented with 3 mmol/l glucose. Then, islets were incubated for

1 h at 37°C. Next, islet groups were incubated first in 1 mmol/l glucose secretion buffer for 1 h, and afterwards in 16 mmol/l glucose secretion buffer for 1 h.

To evaluate insulin paracrine effect on glucagon secretion *ex vivo,* isolated islets were pre-incubated for 1 h at 1 mmol/l glucose "secretion buffer"; then islets were treated with insulin 100 nmol/l (Sigma, USA) in 1 mmol/l glucose "secretion buffer" (control group was treated with vehicle instead of insulin). To evaluate the effect of pharmacological inhibition of IDE on glucagon secretion *ex vivo,* we used the IDE-specific activity inhibitor 6bK (Tocris Bio-techne, USA). Isolated islets were pre-incubated with 10 µmol/l 6bK and afterwards incubated at 1 and 16 mmol/l glucose "secretion buffer" as described above.

The extracellular medium was collected after each incubation, and glucagon and insulin concentration were measured by ELISA as described above. To determine pancreas glucagon and insulin content, after dissection, whole pancreas was incubated overnight in acid-ethanol buffer (1.5% v/v HCl in EtOH) at 4°C and hormones were measured from supernatant by ELISA.

Flow cytometry analysis in isolated islet cells

After islet isolation, islet cells were dispersed by trypsin, isolated islet cells (IICs) were washed with Flow cytometry analysis (FACS) buffer (PBS containing 1% BSA, 1 mmol/l EDTA and 0.01% sodium azide), and then labelled with Live/Dead Fixable Near-IR Dead Cell stain (Invitrogen, USA) according to kit instructions and fixed with 2% paraformaldehyde 10 min at 4 °C. Cells were permeabilised with 0.5% Triton X100 in FACS buffer for 10 min at room temperature, and then incubated for 30 min with blocking solution (2% normal goat serum in FACS Buffer). IICs were incubated for 30 min at 4 °C in dark with antibodies in blocking solution, 1:1,000 rabbit anti-IDE (Millipore, USA) and 1:1,000 mouse anti-glucagon conjugated with Alexa Fluor® 594 (R&D Systems, USA), and then incubated in dark for 30 min at 4 °C in blocking solution with 1:1,000 goat anti-rabbit conjugated with Alexa Fluor® 488 (Invitrogen, USA). Cells were stored in FACS buffer at 4 ºC until spectral flow cytometric analysis. Fluorescence minus one (FMO) controls were used to determine positive staining. See ESM Table 2 for antibodies information.

Cells were acquired on the Aurora Spectral Flow Cytometer (CYTEK) and analyzed using Kaluza software (Beckman Coulter) version 2.1.1.

RNA isolation and qRT-PCR

Islet, liver, kidney, muscle and fat RNA was extracted using TRIZol® Reagent (Thermo Fisher Scientific, USA), according to the manufacturer's instructions. Quantification of

mRNA levels was determined using NanoDrop™ N-D1000 spectrophotometer. These samples were treated with RapidOut DNA Removal Kit (Thermo Fisher Scientific, USA). First strand cDNA was synthesized with iScript™ cDNA synthesis kit (Bio-Rad, USA) as described by manufacturer. qPCR was carried out on equal amounts of cDNA in triplicates for each sample using Maxima Probe qPCR Master Mix (Thermo Fisher Scientific, USA) with corresponding TaqMan® Gene Expression Assays (Applied Biosystems, USA) in a thermal cycler (Lightcycler 480, Roche, Switzerland). Data were normalized with the housekeeping gene *Rpl18* and relative expression was quantified using the comparative $2^{-\Delta\Delta CT}$ method. See ESM Table 1 for primers and TaqMan assays.

Pancreas histomorphometry

Four-month-old mice (A-IDE-KO mice administered tamoxifen at 8 weeks and $Ide^{f/f}$ controls) were euthanized, then their pancreata were dissected, weighed and fixed in 10% neutral buffer formalin overnight at 4°C, embedded into paraffin blocks then cut into 5 µm sections and mounted on glass slides. Staining was performed in two sections per mouse pancreas spaced at least 200 µm apart. To analyze pancreas histomorphometry, sections were incubated with anti-insulin antibody (Abcam, UK) for beta-cell area and anti-glucagon antibody (Abcam, UK) for alpha-cell area, washed, then incubated with HRP-conjugated secondary antibodies, washed and then stained using 3,3′-diaminobenzidine tetrahydrochloride (Sigma, USA) as substrate. Finally, sections were counterstained with hematoxylin as previously reported [13]. Images were acquired using a Nikon Eclipse 90i microscope fitted with a Nikon CCD camera (DSRi1), using a 20X objective with transmitted light. beta-cell area, alpha-cell area and islet number were calculated using Image J 1.52p software (NIH, USA) [12,17]. To analyze alpha cell peripheral location, cells not found in the outer layer of the islet or adjacent to it were counted as "delocalised." See ESM Table 2 for antibodies information.

Pancreas immunostaining

A-IDE-KO and control pancreas sections were stained with the following antibodies diluted in blocking solution (1% BSA, 0.2% normal goat serum in PBS): anti-glucagon (Abcam, UK), anti-IDE (Millipore, USA), anti-Ki67 (Invitrogen, USA), anti-vamp2 (Cell Signaling, USA) and anti-α-synuclein (Santa Cruz Biotechnology, USA). Fluorescent secondary antibodies in blocking solution were used for detection. All antibodies were previously validated by the manufacturer and previous publications [2, 12]. Sections were counterstained with nuclear DAPI staining. Fluorescence images of the sections were acquired using the aforementioned Nikon microscope and camera using a 40X objective.

All pictures were obtained using the same exposure conditions. See ESM Table 2 for antibodies information.

Immunofluorescence intensity of IDE, Vamp2 and α-synuclein were quantified by Image J 1.52p software (NIH, USA) using the following protocol: separated photos of glucagon and Vamp2 were taken using a 40X objective. A specific selection of glucagon-stained area was made by applying a suitable threshold and using the "Create selection" tool within the Image J software on the glucagon image. The selection of glucagon area was transferred into the IDE, Vamp2 or α-synuclein image of the same section, then the intensity of this specific area was quantified using the tool "Integrated Density" within the imaging software, thus yielding a measure of IDE, Vamp2 or α-synuclein intensity/glucagon area. In order to measure the intensity by cell, all cells in images glucagon-stained were quantified with the Image J 1.52p software "cell counter" tool, thus yielding a measure of integrated intensity of Vamp2 staining/alpha-cell number.

Alpha-TC-1.9 cell culture and *Ide* knockdown

Mouse alpha-TC-1.9 cells were obtained from the American Type Culture Collection (ATCC, USA) and they were negative for mycoplasma contamination. Cells were grown at 37.0°C in a 5% CO_2 incubator in DMEM containing 16 mmol/l glucose, 10% heat-inactivated FBS, 15 mmol/l HEPES, 0.1 mmol/l nonessential amino acids, 10 IU/ml penicillin and 10 µg/ml streptomycin. For *Ide* knockdown, 24 h following plating without antibiotic, cells were transfected with siRNA-scramble (siRNA-CTL) or siRNA-*Ide* (Dharmacon, USA) using Lipofectamine 2000 (Invitrogen, USA). Transfection conditions per reaction were as follows: 100 ng siRNA, 500 µl OptiMEM, 10 µl Lipofectamine2000. Solution was incubated at room temperature for 20 min and added to one well. 6 h after transfection 1.5 ml of complete fresh medium was added, then cells were incubated for 48 h.

Alpha-TC-1.9 proliferation studies

To quantify proliferation rates, cells were seeded on coverslips (at least 100,000 cells/coverslip) and incubated with 10 µmol/l BrdU for 6 h. Cells were fixed with 10% formalin for 5 minutes, washed with PBS, immersed in 70% EtOH at 4°C for 30 min; then, cells were immersed in 1 N HCl for 20 min, followed by a washing step with PBS. To prevent non-specific binding, the cells were incubated for 1 h in "blocking solution" (1% BSA, 0.2% normal goat serum in PBS) at room temperature. Staining was performed using monoclonal anti-BrdU rat antibody (Abcam, UK) at 4ºC overnight. The

samples were then incubated with the anti-rat 594 Alexa-fluor-conjugated secondary antibody (Thermoscientific, USA) for 30 min at room temperature and, finally, were mounted onto glass slides using Fluoroshield with DAPI (Sigma, USA). Images were acquired using the aforementioned Nikon microscope and camera with a 40X objective. BrdU positive cells were quantified using the free software Image J (NIH, USA).

To detect the presence of primary cilia in proliferating cells, they were seeded and fixed as described above, after "blocking solution" incubation cells were treated with anti-BrdU antibody (Abcam, UK) for 1 hour and subsequently with anti-α-acetylated α-tubulin (Sigma, USA) antibody at 4°C overnight. The next day the samples were washed with PBS to remove excess primary antibody and incubated with the appropriate Alexa-fluor (Thermofisher, USA) secondary antibodies for 30 minutes at room temperature. Finally, they were mounted with Fluoroshield with DAPI mounting medium (Sigma-Aldrich, USA) for photographing and subsequent analysis. Ciliated and non-ciliated proliferating cells were quantified using the free software Image J 1.52p (NIH, USA). See ESM Table 2 for antibodies information.

<u>Western blotting</u>

Livers from A-IDE-KO mice and siRNA-CTL and siRNA-Ide α-TC1.9 cells were homogenized in lysis buffer (125 mmol/l Tris, pH 6.8, 2% SDS, and 1 mmol/l DTT supplemented with protease and phosphatase inhibitors) and briefly sonicated and centrifuged. Proteins were quantified using the Micro BCA Kit (Thermo Scientific, USA), separated by SDS-PAGE, and then transferred to PDVF Immobilon-P membranes (Millipore, USA). Blots were incubated with the following antibodies and dilutions: anti-GAPDH at 1:20,000 dilution (Millipore, USA), anti-IDE 1:20,000 at dilution (Millipore, USA), anti-glucagon receptor at 1:1,000 dilution (Abcam, UK), anti-Creb at 1:1,000 dilution (Cell Signaling, USA), anti-pCreb at 1:1,000 dilution (Cell Signaling, USA), anti-insulin receptor-ß (IRβ) at 1:1,000 dilution (Cell Signaling, USA), anti-α-synuclein at 1:5,000 dilution (Santa Cruz Biotechnology, USA), anti-acetylated-α-tubulin at 1:10,000 dilution (Sigma, USA), anti α/β-tubulin at 1:5,000 (Cell Signaling, USA), anti-Arl13b at 1:1,000 (NeuroMab, USA). Primary and secondary antibodies were prepared in WB buffer (0.5% non-fat milk, 0.01% Tween, PBS). All antibodies were previously validated by the manufacturer and previous publications [11, 13]. See ESM Table 2 for antibodies information.

<u>Ca^{2+} signaling experiments</u>

Freshly isolated islets were left to recover in a Krebs-Ringer HEPES-buffered solution (pH = 7.35; 5 mmol/l glucose) for 2 hours in the incubator. After recovery, islets were

loaded with 2.5 µM Fluo-4-AM (Invitrogen, USA) for at least 2 hours at room temperature and then, transferred to a perfusion chamber coupled to a thermostatic bath that was mounted on a Zeiss LSM 510 laser confocal microscope. The Ca^{2+} probe was excited at 488 nm, while emission was collected with a band-pass filter at 505–530 nm. Individual cells within intact islets were monitored in a thin optical section across the islet (8-10 µm) [53]. Islets were perfused at 35ºC with the above-mentioned solution at either 1 or 16 mmol/l glucose. Beta-cells were identified by their typical Ca^{2+} response to glucose characterized by either a sustained Ca^{2+} elevation or a transient followed by oscillations as well as their characteristic synchronic behavior due to cell coupling [47,48]. Alpha-cells were categorized according to their characteristic pattern consisting of spontaneous Ca^{2+} oscillations at low glucose levels [47] and the ability of high glucose to decrease Ca^{2+} signaling [49]. Since alpha-cells are not functionally coupled with beta-cells [47], we excluded for the analysis those alpha-cells that exhibited signal contamination from adjacent beta-cells. To determine the proportion of cells that exhibit complete suppression of Ca^{2+} signaling at 16 mmol/l glucose or still displayed Ca^{2+} activity, Ca^{2+} oscillations were analyzed in the last 6 minutes of the recording at high glucose concentration to allow complete equilibration of perfused media in the islet chamber. We used a contingency Fisher's exact test to analyze differences in the proportion of blocked and active cells between control and A-IDE-KO groups with a level of significance $p<0.05$.

IQR method for outlier recognition

We have applied this method for identifying outliers in the qRT-PCRs, setting up a “fence” outside of Q1 (first quartile, 25th percentile) and Q3 (third quartile, 75th percentile). Any values that fall outside of this fence were considered outliers. To build this fence we took 1.5 times the IQR and subtracted this value from Q1; then, we took 1.5 times the IQR and we added this value to Q3 (Q1-1.5*IQR; Q3+1.5*IQR; being IQR=Q3 – Q1). This resulted in the minimum and maximum fence posts that were used to compare each value to. Any values that were less than Q1-1.5*IQR or more than Q3+1.5*IQR were considered outliers.

Primer/Target gene	Sequence (5`-3`)/Taqman assay
Gapdh_R	GATG GCAT GGA CTG TGG TCA T
Gapdh_F	CGT GGA GTC TAC TGG TGT CTT
Flox_IDE_R	CTC AGG GAT ACA ATG CGT GC
Flox_IDE_F	AAC TGC CAC CTG TCC AAT CC
GcgCreER_F	GCC AGT CAC TTG GGA TGT ACA
GcgCreER_KI_R	AGC CCC TTG TTG AAT ACG CT
GcgCreER_WT_R	CCA GGT GGT CAT GTC TTC TGT
Ngn3	Mm00437606_s1
Arx	Mm00545903_m1
Mafb	Mm00627481_s1
Gcg	Mm00801714_m1
Snap25	Mm01276446_m1
Syntaxin 1A	Mm00444008_m1
Vamp-2	Mm01325243_m1
RPL18_R	AGC CTT GAG GAT GCG ACT C
RPL18_F	AAG ACT GCC GTG GTT GTG G

ESM Table 1: DNA sequences of primers and references of TaqMan

Product	Catalogue #	Company	Working dilution
6bk	5402	Tocris Bio-techne,USA	10 µmol/l
Ultra Sensitive Mouse Insulin ELISA Kit	90080	Crystal Chem, USA	
Mouse insulin ELISA kit	10-1247-01	Mercodia, Sweden	
Glucagon ELISA kit	10-1281-01	Mercodia, Sweden	
anti-insulin guinea pig antibody	ab 7842	Abcam, USA	1:100 IHC
anti-glucagon mouse antibody	ab 10988	Abcam, USA	1:1,000 IHC
Peroxidase AffiniPure Donkey Anti-Guinea pig IgG (H+L)	706-036-148	Jackson Laboratories, USA	1:1,000 IHC
anti-Mouse IgG HRP Linked F(ab')2 Fragment	NA 9310 V	Millipore, USA	1:5,000 IHC, WB
Peroxidase AffiniPure Donkey Anti-Rabbit IgG (H+L)	705-035-147	Jackson Laboratories, USA	1:20,000 WB
anti-IDE rabbit antibody	AB 9210	Millipore, USA	1:2,000 IF
anti-IDE rabbit antibody	AB 9210	Millipore, USA	1:20,000 WB
anti-IDE rabbit antibody	AB 9210	Millipore, USA	1:1,000 FACS
anti-Ki67 antibody	MA5-14520	Invitrogen, USA	1:100 IF
anti-VAMP-2 rabbit antibody	13508S	Cell signaling, USA	1:1,000 IF, WB
anti-α-synuclein antibody	sc-12767	Santa Cruz,USA	1:100 IF
anti-α-synuclein antibody	sc-12767	Santa Cruz,USA	1:5,000 WB
Alexa fluor ® 488 F(ab`)2 fragment to goat anti-rabbit IgG	A 11070	Thermosientific, USA	1:1,000 IF, FACS
Alexa fluor ® 594 goat anti-mouse IgG	A 11005	Thermosientific, USA	1:1,000 IF
Alexa fluor ® 488 goat anti-mouse IgG	A 11001	Thermosientific, USA	1:1,000 IF
Alexa fluor ® 594 goat anti-rabbit IgG	A 11012	Thermosientific, USA	1:1,000 IF
anti-glucagon conjugated with Alexa Fluor® 594	IC1249T	R&D Systems, USA	1:1,000 FACS
anti-BrdU rat antibody	ab6326	Abcam, UK	1:1,000 IF
anti-GAPDH mouse antibody	MAB374	Millipore, USA	1:20,000 WB
anti-glucagon receptor rabbit antibody	ab10988	Abcam, UK	1:1,000 WB
anti-CREB rabbit antibody	9197S	Cell Signaling, USA	1:1,000 WB
anti-pCREB rabbit antibody	9198S	Cell Signaling, USA	1:1,000 WB
anti-insulin receptor-ß (IRβ) rabbit antibody	3025S	Cell Signaling, USA	1:1,000 WB
anti-acetylated-α-tubulin mouse antibody	T7451	Sigma, USA	1:1,000 IF
anti-acetylated-α-tubulin mouse antibody	T7451	Sigma, USA	1:10,000 WB
anti α/β-tubulin rabbit antibody	2148S	Cell Signaling, USA	1:5,000 WB
anti-Arl13b mouse antibody	N295/66	NeuroMab, USA	1:1,000 WB

ESM Table 2: Key resources and working conditions. *Immunohistochemistry (IHC); Western-blot (WB); Immunofluorescence (IF), Flow cytometry analysis (FACS).*

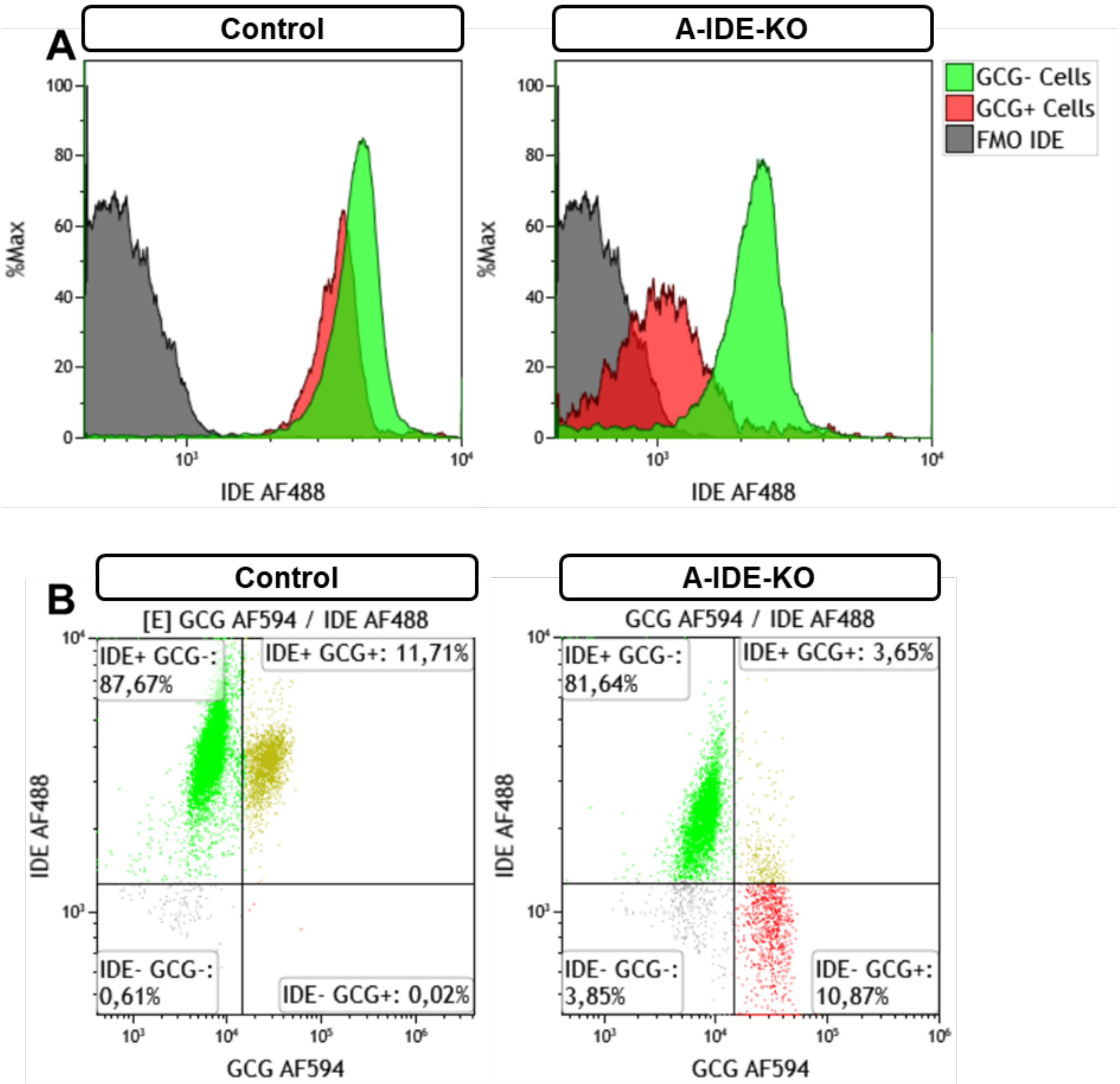

ESM Fig. 1. Flow Cytometry Analysis of Isolated Islet Cells Labeled with anti-IDE and anti-glucagon (GCG) antibodies. *A: Overlaid histograms of fluorescence intensity from IDE labeling in Control and A-IDE-KO isolated islet cells (IICs). In Control-IICs, histograms of both glucagon negative (GCG-) and glucagon positive (GCG+) are overlapped and far away from FMO histogram, meaning that all cells are positive to IDE. In A-IDE-KO-IICs, the histogram of GCG+ overlaps with the FMO and is far away from GCG- histogram, meaning that most of GCG+ cells are negative to IDE staining B: Dot plots facing fluorescence intensity from GCG vs. IDE labeling show in Control-IICs that ~12% of total islet cells are GCG+/IDE+; meanwhile in A-IDE-KO-IICs, ~11% of total islet cells are GCG+/IDE-.*

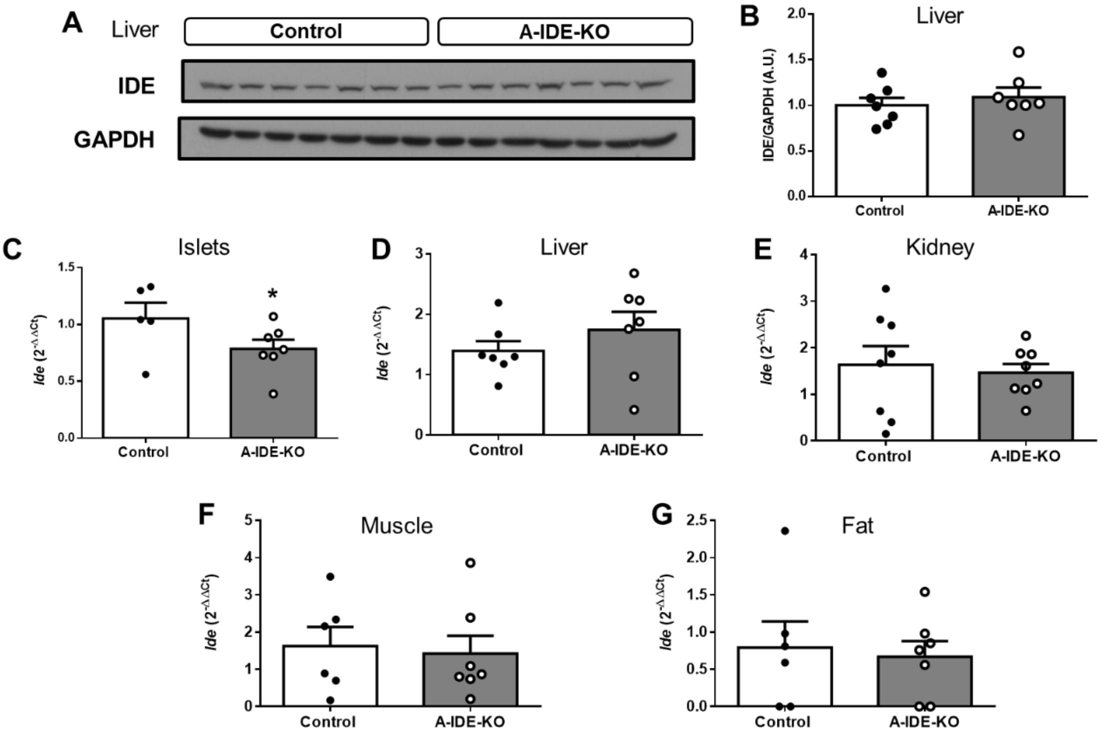

ESM Fig. 2: IDE expression in other tissues involved in insulin clearance and glucose metabolism. *A: Representative western blot of IDE protein in the liver of A-IDE-KO mice. B: Western blot quantification of liver IDE protein from A-IDE-KO mice. C-G: Ide gene expression from islets, liver, kidney, muscle and fat. *p<0.05.*

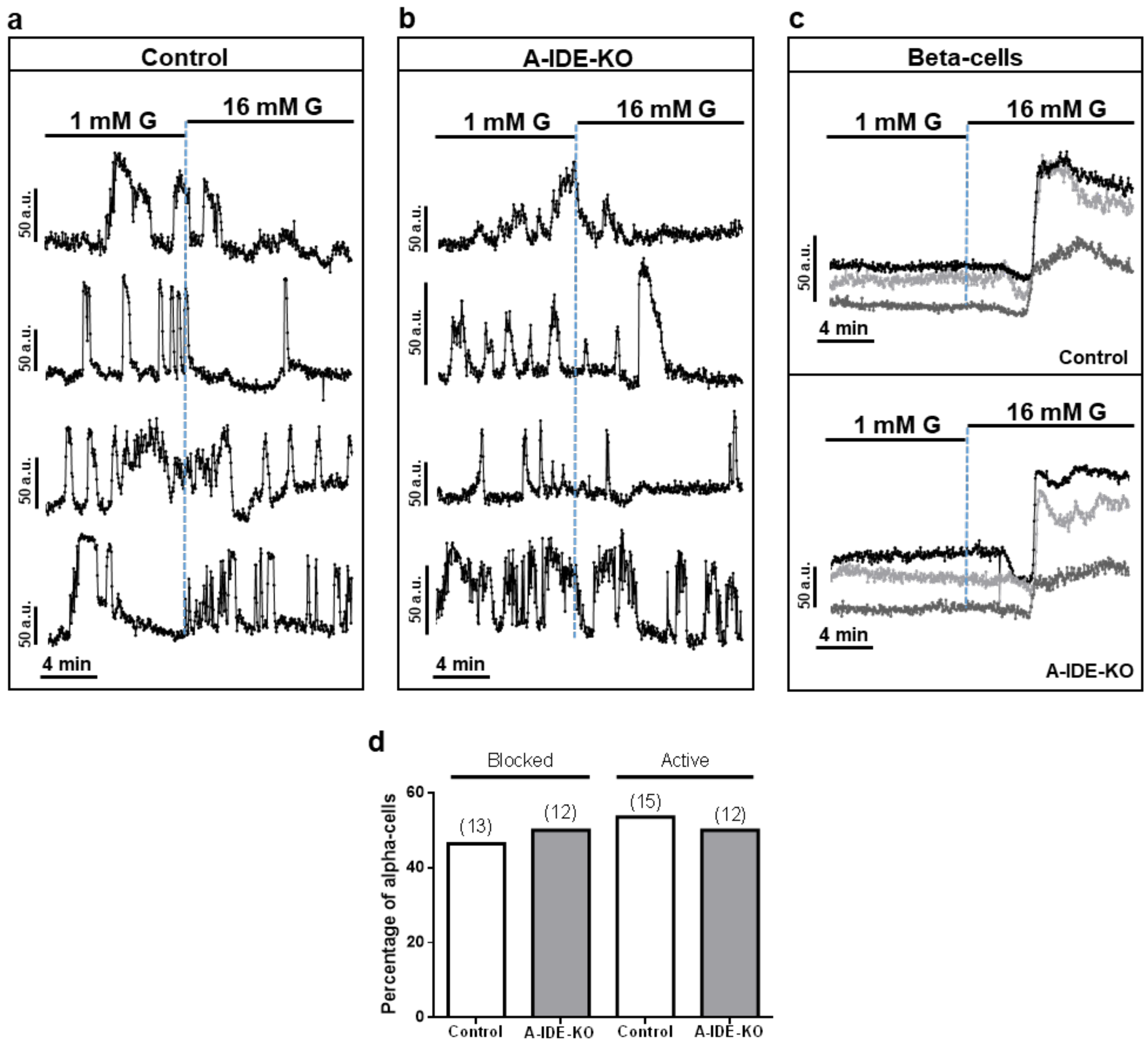

ESM Fig. 3: Ca^{2+} signaling in control and A-IDE-KO islet cells. *A, B: Representative recordings of several alpha-cell patterns in different control (A) and A-IDE-KO (B) islets. Alpha-cells displayed a characteristic response consisting of spontaneous Ca^{2+} oscillations at low glucose levels. Exposure of islets to 16 mmol/l glucose (G) resulted in cells showing a complete suppression of Ca^{2+} signals or in cells that remained active, but generally with a lower frequency in Ca^{2+} oscillations. C: Representative recordings of several beta-cells within the same control or A-IDE-KO pancreatic islets. Beta-cells usually displayed a sustained Ca^{2+} rise in response to 16 mmol/l glucose (or a transient followed by oscillations) and the characteristic synchronic behavior due to cell coupling. D: Percentage of alpha-cells that were completely blocked or exhibited Ca^{2+} signaling activity at 16 mmol/l glucose. The number of cells for each condition are shown in each column. A contingency Fisher's exact test was performed, showing non-significant differences in the proportion of blocked and active cells between control and A-IDE-KO groups. Alpha-cell Ca^{2+} signals were analyzed in 28 cells from 10 control islets and 24 cells from 11 A-IDE-KO islets. Beta-cell Ca^{2+} signals were recorded in at least 73 cells from 13 control islets and 56 cells from 12 A-IDE-KO islets. Three mice per group were analyzed.*

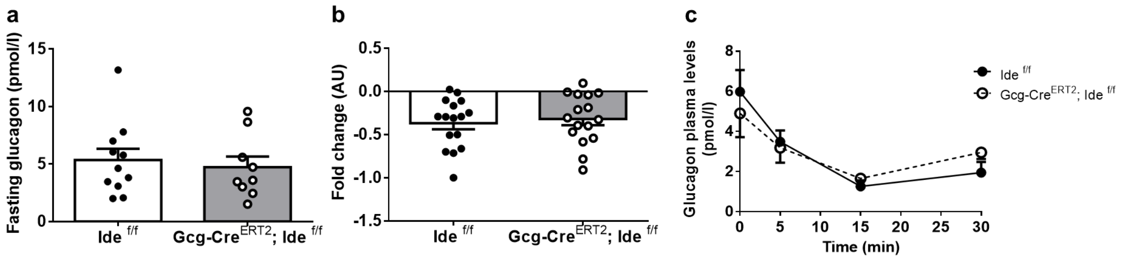

ESM Fig. 4: Characterization of glucagon secretion in Gcg-Cre^{ERT2}; Ide$^{f/f}$ mice not treated with tamoxifen. *A: Fasting plasma glucagon levels. B: Fold change of glucagon secretion after glucose stimulation (from 1 mmol/l to 16 mmol/l glucose) (N=16 islet secretion groups per condition). C: Plasma glucagon levels in response to glucose challenge after fasting (16 h). N=7-9 mice per group.*
